# Doxycycline-Associated Dual Cutaneous Adverse Reaction to the Drug (CARD): Case Report of Concurrent Photosensitivity and Morbilliform Exanthem to Doxycycline

**DOI:** 10.7759/cureus.11546

**Published:** 2020-11-18

**Authors:** Joanne S Jacob, Philip R Cohen

**Affiliations:** 1 Medicine, Baylor College of Medicine, Houston, USA; 2 Dermatology, San Diego Family Dermatology, National City, USA

**Keywords:** adverse, antibiotics, cutaneous, dermatitis, doxycycline, drug, medication, morbilliform, photosensitivity, reaction

## Abstract

Antibiotics have been observed to cause drug-induced reactions. These can include a cutaneous adverse reaction to the drug (CARD) such as photosensitivity. A 51-year-old woman initiated doxycycline monohydrate for rosacea. Within nine days, she developed two different, simultaneous skin rashes: a phototoxic reaction and a morbilliform drug eruption. The medication was stopped; topical and oral corticosteroids were initiated. Within two weeks, her rashes resolved. Common cutaneous adverse reactions to doxycycline include photosensitivity and morbilliform exanthem. Less common skin side effects include bullous eruptions, lupus-like eruptions, pigmentary disorders, and vasculitis. Albeit uncommon, doxycycline-associated dual CARD - such as the photosensitivity and morbilliform exanthem - may occur.

## Introduction

Systemic antibiotics are commonly prescribed for the management of not only infections but also other conditions. However, antibiotics can be associated with numerous side effects. These include not only systemic reactions to the medication but also cutaneous adverse reaction to the drug (CARD) [1]. Doxycycline is a tetracycline derivative. It can be used in the management of bacterial skin infections like methicillin-resistant Staphylococcus aureus. Also, it is a mainstay therapy for cutaneous disorders such as acne and rosacea [2].

Photosensitivity has been associated with several drugs [1,3]. A morbilliform exanthem has also been observed in patients who are allergic to medication [4]. Features of a woman who started doxycycline monohydrate for the treatment of rosacea and developed a concurrent cutaneous adverse reaction to the antibiotic is described.

## Case presentation

A 51-year-old woman had a history of rosacea of several years duration. She had been evaluated and treated by numerous prior physicians. Her recent management included systemic erythromycin 500mg twice daily and topical 0.75% metronidazole gel; however, her symptoms and clinical appearance had worsened. Her management was changed. The erythromycin was discontinued, and doxycycline monohydrate 100 mg twice daily was started. Within nine days, she developed two different, yet concurrent, skin rashes to the doxycycline.

**Figure 1:**
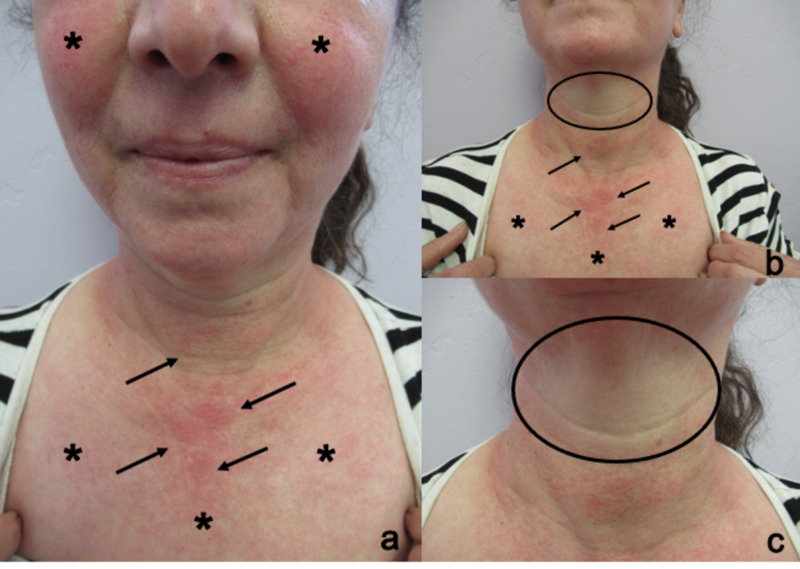
Phototoxic drug reaction to doxycycline in sun-exposed areas A 51-year-old woman developed doxycycline-related phototoxic reaction. Views of face (a and b), neck (a, b, and c) and chest (a, b, and c) show telangiectasias (black asterixis), and pruritic macular erythema (black arrows) in the areas exposed to the sun. However, there is sparing on the skin between the chin and neck (within the black oval) that was not sun-exposed.

**Figure 2:**
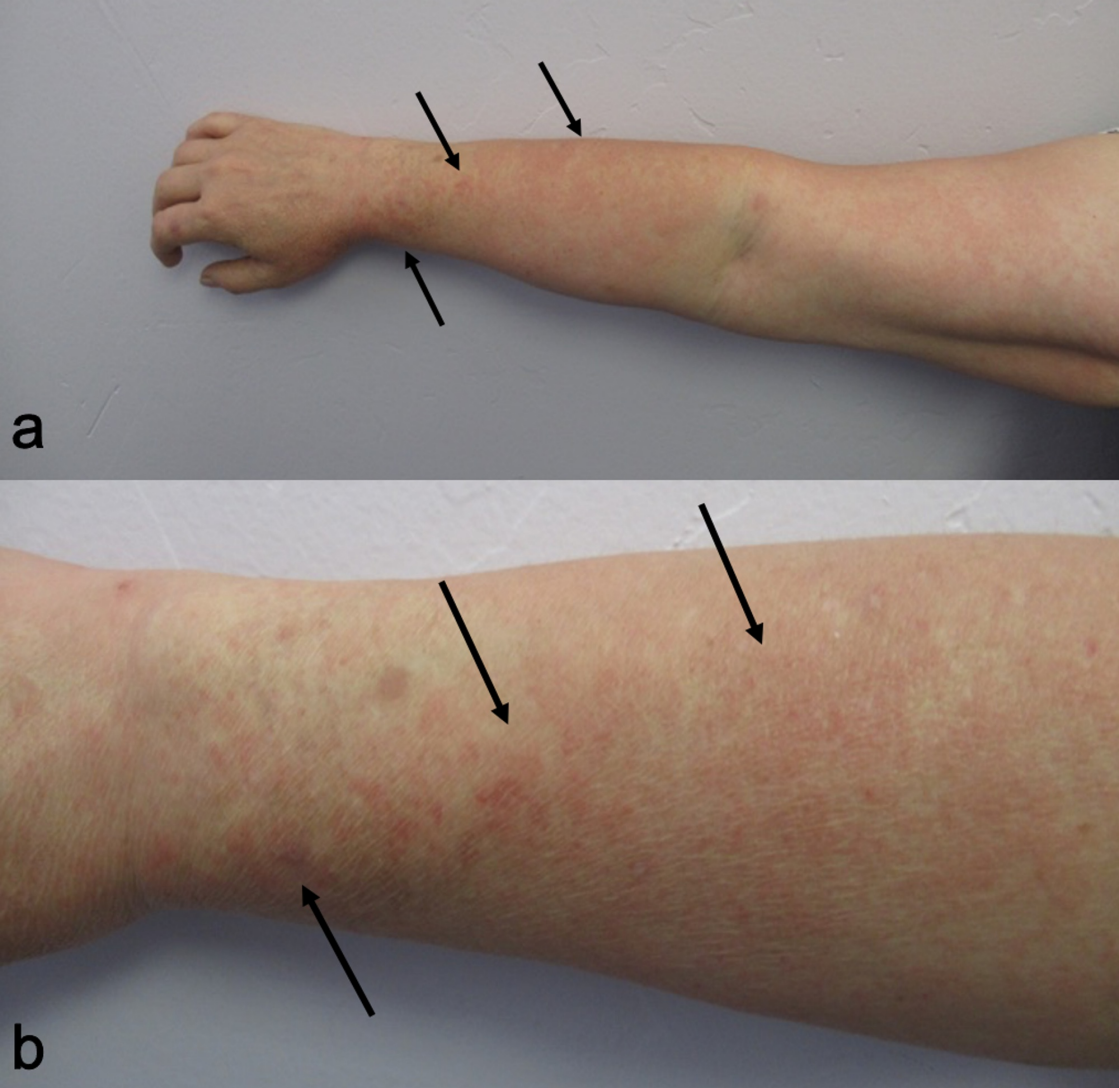
Doxycycline-associated phototoxic cutaneous adverse reaction to the drug (CARD) Distant (a) and closer (b) views of phototoxic drug reaction to doxycycline in a 51-year-old woman presenting as pruritic, macular erythema (black arrows) on the sun-exposed areas of the right forearm that were not covered by her shirt sleeve.

One of the skin lesions was limited to sun-exposed areas of the face and neck; it also affected the forearms corresponding to areas not covered by her shirt sleeves. There was pruritic macular erythema with telangiectasias on her face and chest. The lesions on her cheeks were not only secondary to rosacea but also exacerbated by sun exposure. There were also erythematous patches on her chest with distinctive sparing of the area between chin and neck, which had not exposed to the sun (Figure 1). Also, there was macular erythema on her forearms (Figure 2).

**Figure 3:**
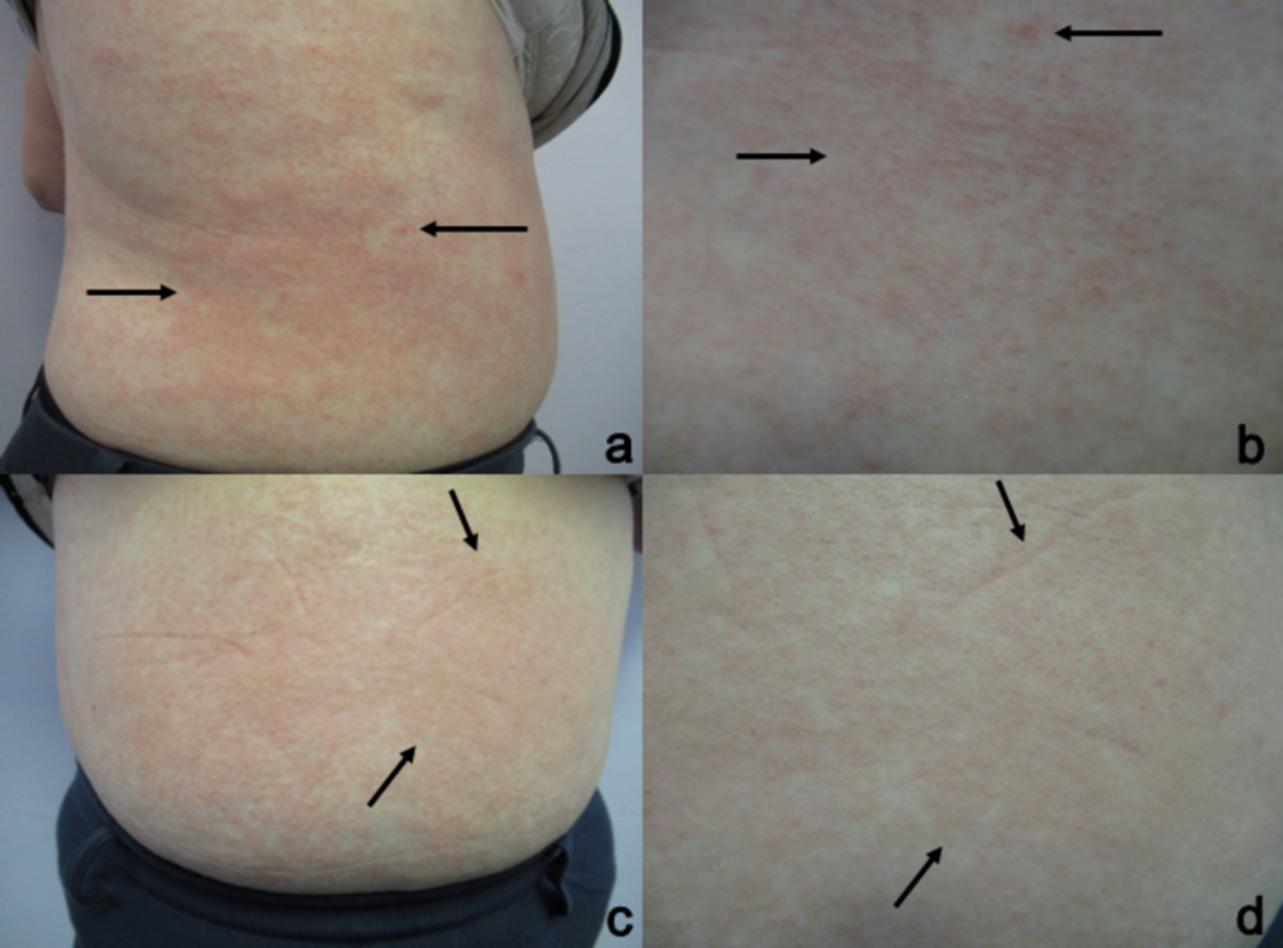
Doxycycline-related morbilliform drug eruption Cutaneous adverse reaction to the drug (CARD) that developed nine days after beginning treatment with doxycycline. The right flank (a and b) and the abdomen (c and d) show distant (a and c) and closer (b and d) views of extremely pruritic morbilliform macules and confluent patches (black arrows) on non-sun exposed skin.

The second-skin rash was extremely pruritic. It consisted of morbilliform macules and confluent patches on non-sun exposed skin. The exanthem was present on her flanks and abdomen (Figure 3).

Management of the new CARD included stopping the doxycycline. The topical metronidazole gel was also discontinued. Oral antihistamines (loratadine 10mg in the morning and hydroxyzine 25mg in the evening) were started. A short course of oral prednisone was started: 60 mg each morning for six mornings followed by 40mg each morning for four mornings and then 20mg each morning for two mornings. Also, triamcinolone 0.1% cream was applied twice daily to all affected areas.

Follow-up after one week of treatment showed significant improvement of the pruritis and skin lesions. After two weeks of therapy, all of her symptoms and lesions had resolved. She resumed her topical rosacea regimen with twice daily application of the metronidazole gel.

## Discussion

Tetracyclines are a class of antibiotics that include demeclocycline, doxycycline, minocycline, and tetracycline. The mechanism of action is binding to the 30s ribosomal subunit of bacteria and inhibiting bacterial protein synthesis when treating infection [2]. However, when the drug is used in acne and rosacea, the therapeutic effect may also be secondary to its anti-inflammatory properties. Systemic and cutaneous adverse reactions may occur in patients treated with tetracyclines.

Doxycycline CARD includes photosensitivity; also, patients may develop a morbilliform exanthem. Less common skin side effects include bullous eruptions, lupus-like eruptions, pigmentary disorders, and vasculitis [5-8]. The development of simultaneous or multiple doxycycline-induced CARD is unique; indeed, we are not aware of a previous description of this phenomenon. Photosensitivity to drugs includes photoallergic and phototoxic reactions. The occurrence of these varies. The incidence of photoallergic reactions is low, and phototoxic reactions are high (Table 1) [1,3,9-11].

**Table 1:** Occurrence of drug-associated photoallergic and phototoxic reactions

Characteristic	Photoallergic reactions	Phototoxic reactions
Incidence	Uncommon	Common
Prior exposure to drug	Required	Not required
Dose-dependence	No; can occur with low doses	Yes; increases with higher dose

The clinical characteristics of photosensitivity drug reactions are summarized in Table 2 [1,3,9-11]. Photoallergic reactions appear as eczematous lesions that can occur on both sun-exposed and non-exposed skin. In contrast, phototoxic reactions typically present as edematous patches on sun-exposed skin; vesicle and bullae formation may occur.

**Table 2:** Clinical presentation of drug-associated photoallergic and phototoxic reactions

Characteristic	Photoallergic reactions	Phototoxic reactions
Onset	Does not occur following initial exposure	Occurs following initial exposure
Time of onset	Occurs one to three days after initiation of drug	Occurs within minutes to hours after initiation of drug
Appearance	Dermatitis	Sunburn-like inflammation
Distribution	Starts on sun-exposed areas and spreads throughout entire body	Only sun-exposed areas
Morphology	Eczematous lesions with pruritis	Erythematous and edematous lesions; overlying blisters may occur

The histology of photoallergic reactions shows spongiosis of the epidermis and infiltration of the dermis with lymphocytes. In contrast, the pathologic changes of phototoxic reactions show necrosis of the epidermis and infiltration of the dermis with lymphocytes, macrophages, and neutrophils [9,10].

The management of photosensitivity reactions usually involves stopping the causative agent. Resolution of the reaction with pigmentary changes may occur; however, this is uncommon in photoallergic reactions and common in phototoxic reactions [9,11]. Reduction of ultraviolet radiation exposure, in addition to the use of sunscreen and protective clothing, can decrease the occurrences of the CARD [10].

In addition to tetracyclines, other antibiotics are also associated with photosensitivity (Table 3) [11-20]. Fluoroquinolones, sulfonamides and tetracyclines are the most common culprits [13,14,16-20]. However, cephalosporins and penicillins have also been rarely observed to cause photosensitivity [12,15].

**Table 3:** Systemic antibiotics associated with photosensitivity

Class of drug	Drug	References
Cephalosporins	Ceftazidime	[12]
Fluoroquinolones	Ciprofloxacin, enoxacin, levofloxacin, ofloxacin, sitafloxacin, sparfloxacin	[13,14]
Penicillins	Amoxicillin	[15]
Sulfonamides	Dapsone, trimethoprim-sulfamethoxazole	[16, 17]
Tetracyclines	Demethylchlortetracycline, doxycycline, minocycline, tetracycline	[18-20]

Morbilliform exanthem, often pruritic, is a frequent presentation of drug allergy that occurs about four to 21 days after starting a new medication. This type of skin reaction requires discontinuation of the agent. It will subsequently resolve either spontaneously or after treatment with systemic antihistamines and/or corticosteroids applied topically or given systemically or both. Repeat administration of the causative agent will result in recurrence of CARD [4].

Our patient developed a dual doxycycline-associated CARD: photosensitivity and morbilliform exanthem. Both skin side-effects coincided. Similarly, both CARD promptly resolved after stopping the doxycycline and initiating topical and system interventions to alleviate the drug-associated symptoms and lesions.

## Conclusions

CARD is common. Antibiotics are frequently associated with a CARD. These include not only a generalized skin eruption but also photosensitivity. Classes of antibiotics associated with photosensitivity include cephalosporins, fluoroquinolones, penicillins, sulfonamides, and tetracyclines. We observed a woman who developed photosensitivity and a morbilliform exanthem to doxycycline after the drug was prescribed to treat her rosacea. The photosensitivity presented as pruritic macular erythema on sun-exposed areas of the face, neck, and forearms. The morbilliform exanthem was observed as erythematous macules and patches on the flanks and abdomen. Both of the CARDs to doxycycline resolved within two weeks after initiation of oral antihistamines, oral prednisone, and topical triamcinolone. Albeit seldom, concurrent CARD may occur; in our patient, doxycycline-associated dual CARD - photosensitivity and morbilliform exanthem - occurred.
